# A half-century of VEGFA: from theory to practice

**DOI:** 10.1172/JCI184205

**Published:** 2024-08-01

**Authors:** Susan E. Quaggin

**Affiliations:** 1 Feinberg Cardiovascular & Renal Research Institute, and; 2 Division of Nephrology, Department of Medicine, Northwestern University, Feinberg School of Medicine, Chicago, Illinois, USA.

## Identifying a blood vessel growth factor

The discovery of the potent angiogenic factor VEGFA and the subsequent studies that led to the development and successful translation of VEGF inhibitors into the clinic illustrate the invaluable role physician-scientists play in advancing human health. In 1972, pediatric surgeon and cancer pioneer Judah Folkman published an editorial in the *New England Journal of Medicine*, where he introduced the concept of antiangiogenic therapy to slow the growth of solid tumors (1). Based on work performed in his own laboratory and clinical observation, he had isolated a tumor angiogenic factor (TAF) that promoted endothelial cell proliferation and neovascularization. Folkman, in collaboration with pathologist Hal Dvorak, also recognized the importance of vascular permeability with ensuing extravascular clotting to lay down the necessary tumor stroma to support angiogenesis and tumor growth and metastasis, and the two began their quest to identify the vascular permeability factor (VPF) (2). To accomplish this, tumor cells were cultured in serum-free media and supernatants were tested for the presence of a vascular permeability–inducing factor(s) secreted by the cells (3, 4). They soon found that several tumor types possessed this vascular permeability–promoting activity, and they went on to successfully isolate the factor, demonstrating that VPF was 50,000 times more potent than histamine on a molar basis.

Following the discovery of VPF, Connolly et al. published a study in the *JCI* (5), where they reported the unexpected result that VPF — isolated from guinea pig line 10 tumor cells — not only enhanced vascular permeability, but also promoted endothelial cell proliferation and the growth of new blood vessels when injected into healing rabbit bone grafts or rat corneas. In their landmark study, they demonstrated high-affinity binding of VPF to endothelial cells, vascular selectivity of the growth factor response in vitro, the ability of VPF to promote blood vessel growth in vivo, and provided evidence that a receptor for VPF was present on endothelial cells. The discovery that, in addition to promoting vascular permeability, VPF had a direct effect on endothelial cell migration and proliferation and could induce angiogenesis in vivo (5–7) led to the realization that VPF was Dr. Folkman’s long sought after tumor angiogenesis factor (TAF).

In the same year as Connolly et al.’s paper, Napoleone Ferrara published a report (7) describing the purification of a proangiogenic factor secreted by bovine folliculo-stellate cells (FCs) and successful isolation of both bovine and human complementary DNA clones from FCs and hL60 leukemia cells, respectively. The gene product was named vascular endothelial growth factor (VEGF/VEGFA), and sequence comparison confirmed VEGF and VPF were one and the same.

With rapid molecular advances and sequence in hand, together with the prediction that stopping or inhibiting tumor blood vessel growth had the potential to stop tumor vessels in their tracks, the therapeutic race was on! Simultaneously, the scientific community set about to understand the role of this potent blood vessel growth factor in multiple developmental and disease states.

## A finely tuned system

In 1996, two independent groups reported the surprising result that haploinsufficiency of *Vegfa* in mice was incompatible with life due to embryonic lethality from severe cardiovascular defects (8, 9). This was the first report of a non-imprinted gene causing embryonic lethality in the haploinsufficient state and underscored the importance of exquisite regulation of VEGFA dose in physiologic settings. This observation has been borne out in many subsequent preclinical studies and in patients.

The excitement of molecular characterization of such a potent and clearly important factor led to many more questions, including whether VEGFA plays a role in other diseases that involve the vasculature besides cancer. Subsequent work in the field described altered levels of VEGFA in tissues and compartments affected in diseases characterized by vascular abnormalities such as diabetic proliferative retinopathy, inflammatory skin diseases, pulmonary diseases, myocardial infarction, and peripheral vascular disease (10–15). These observations led to the possibility of intervening in diseases by delivering VEGFA when new blood vessel growth is desired or inhibiting VEGFA when blocking pathologic angiogenesis is needed.

Zhang et al. (16) examined the role of VEGFA in the setting of ischemic stroke — an often irreversible and severe disease that is associated with long-lasting morbidity and mortality. In a rodent model of ischemic stroke, they demonstrated that delivery of recombinant VEGFA, 48 hours after infarct, increased revascularization and improved neurologic outcome, whereas administration of VEGFA in the acute setting increased blood-brain-barrier leak and elevated the risk of transformation to hemorrhagic stroke. This study highlighted the potential benefits and risks of modulating VEGF activity and highlighted the narrow safety window to achieve benefit versus harm.

## From lab to clinic

In the decade following the discovery of VEGFA, inhibition of VEGFA in animal models of cancer demonstrated improved outcomes (17). At Genentech, Napoleone Ferrara, the physician-scientist who had first identified the gene sequence of VEGFA, led the studies needed for the development of the VEGF inhibitor known as bevacizumab, a monoclonal antibody against VEGFA.

In 2004, positive results from the first phase III trial of bevacizumab in humans led to its approval by the FDA as first-line treatment for patients with metastatic colorectal cancer (18). While there were clearly clinical benefits, there were also disappointments with recognition that escape and/or resistance to antiangiogenesis therapies was a common event in tumors. Indeed, over many years, clinicians had recognized that the antiangiogenic potential of many traditional chemotherapeutic agents and approaches was only transient and followed by ultimate escape and recovery of the tumor vessels.

To circumvent this resistance and improve the antiangiogenic benefits of chemotherapy, Robert Kerbel proposed combining low doses of an anti-VEGF agent targeting the VEGFA receptor Flk1/VEGFR2 (DC101) and a chemotherapeutic agent, vinblastine, in hopes of inhibiting VEGFA’s ability to promote recovery and/or survival of endothelial cells following damage from chemotherapy and/or radiation. In 2000, his group showed that this combination led to full and sustained regression of large tumors in a neuroblastoma xenograft model without increase in toxicity, supporting the model that adjuvant treatment with anti-VEGF agents may benefit multiple cancer treatment regimens (19).

## An unanticipated, rare side effect of inhibiting VEGF

Coincident with the emergence of anti-VEGF therapies in clinical use, clinicians began to identify and report adverse effects of these inhibitors in some patients. In my own field of nephrology, we observed an increase in consults for high-grade hypertension, proteinuria, and acute kidney injury in patients receiving anti-VEGF agents. Occasionally, patients also demonstrated thrombocytopenia and hemolytic anemia, and schistocytes on their peripheral blood smears — a clinical triad defining thrombotic microangiopathy (TMA), which is a heterogeneous group of disorders caused by microthrombi in small arteries and capillaries.

Simultaneously, we and others had been intrigued by the reports of high levels of VEGFA expression in podocytes, a specialized glomerular epithelial cell in the kidney that, together with the adjacent fenestrated endothelial cell and intervening glomerular basement membrane, constitutes the glomerular filtration barrier. Hal Dvorak and colleagues had shown podocytes expressed higher levels of VEGFA compared with most other cell types during development (20). Furthermore, unlike many other vascular beds and vascular-adjacent cells, podocytes continued to express VEGFA, albeit at lower levels, in the adult kidney, suggesting that it was important for glomerular barrier integrity (21). With our ability to selectively knock out genes in podocytes, we collaborated with Ferrara and András Nagy to generate an allelic series of mice that expressed varying levels of *Vegfa* in their podocytes (22).

We readily observed that any change in the level of *Vegfa* expression had major consequences for kidney structure and function, with unique phenotypes observed for each dosage change in *Vegfa*. While the gene was deleted from podocytes, the glomerular structural and functional defects were primarily driven by changes in the adjacent glomerular endothelial cells, highlighting the cross-talk between ligand-producing podocytes and VEGF receptor–expressing endothelial cells. Most relevant for patients, we noted that *Vegfa* haploinsufficiency in podocytes led to marked endotheliosis, a phenotype of glomerular endothelial cells that is pathognomonic for TMA and the same glomerular injury observed in patients receiving anti-VEGF agents (22). To extend the work beyond developmental stages, we showed that podocyte-selective deletion of *Vegfa* from fully mature glomeruli led to a thrombotic microangiopathy phenotype too, supporting a causal on-target effect of anti-VEGF agents and TMA in susceptible patients (23).

At the same scientific meeting where our group first presented the mouse TMA phenotype, Ananth Karumanchi presented the exciting discovery of the soluble VEGF receptor 1, FLT1, as a circulating inhibitor of VEGFA. He described the production of placental FLT1 in patients with preeclampsia, a condition characterized by glomerular endotheliosis and TMA. The phenotypic similarities between the proof-of-concept mouse model, the patients receiving anti-VEGF agents, and patients with preeclampsia, led to a wonderful collaboration and many shared discussions. The two stories were subsequently published together in the same issue of the *JCI* (22, 24).

Almost a quarter of a century later, there are now several hundred cases of VEGF inhibitor–induced TMA reported in the literature and this complication is recognized as a class effect that can occur with any drug that targets the VEGFA ligand or its receptor. However, only a small subset of patients receiving the drug develop clinically apparent TMA, suggesting that other predisposing factors contribute. Genetic variants linked to other forms of TMA, such as genes encoding complement regulatory proteins and ADAMTS13, may potentially act as a “second hit” that contributes to disease (25, 26).

## Concluding remarks

Although VEGFA was initially identified because of a surgeon’s clinical observation about the close association of blood vessel and tumor growth, antiangiogenesis therapies have not been as effective in cancer treatment as initially hoped. However, these fundamental clinically driven discoveries led to the development of bevacizumab and other anti-VEGFA agents, which are now the gold standard of care for the treatment of vascular pathologies of the eye, including the wet form of age-related macular degeneration, macular edema, and proliferative retinopathy associated with diabetes. In addition, opportunities to harness the powerful biologic effects of VEGFA with improved delivery and cell-targeted approaches hold promise for future therapies. The story of VEGFA is not yet over.

## Version 1 08/01/2024

Electronic publication

## References

[1] Folkman J (1971). Tumor angiogenesis: therapeutic implications. N Engl J Med.

[2] Dvorak HF (1995). Vascular permeability factor/vascular endothelial growth factor, microvascular hyperpermeability, and angiogenesis. Am J Pathol.

[3] Senger DR (1986). A highly conserved vascular permeability factor secreted by a variety of human and rodent tumor cell lines. Cancer Res.

[4] Senger DR (1983). Tumor cells secrete a vascular permeability factor that promotes accumulation of ascites fluid. Science.

[5] Connolly DT (1989). Tumor vascular permeability factor stimulates endothelial cell growth and angiogenesis. J Clin Invest.

[6] Keck PJ (1989). Vascular permeability factor, an endothelial cell mitogen related to PDGF. Science.

[7] Leung DW (1989). Vascular endothelial growth factor is a secreted angiogenic mitogen. Science.

[8] Carmeliet P (1996). Abnormal blood vessel development and lethality in embryos lacking a single VEGF allele. Nature.

[9] Ferrara N (1996). Heterozygous embryonic lethality induced by targeted inactivation of the VEGF gene. Nature.

[10] Apte RS (2019). VEGF in signaling and disease: beyond discovery and development. Cell.

[11] Folkman J (1995). Angiogenesis in cancer, vascular, rheumatoid and other disease. Nat Med.

[12] Khurana R (2005). Role of angiogenesis in cardiovascular disease: a critical appraisal. Circulation.

[13] Papaioannou AI (2006). Clinical implications for vascular endothelial growth factor in the lung: friend or foe?. Respir Res.

[14] Perez-Gutierrez L, Ferrara N (2023). Biology and therapeutic targeting of vascular endothelial growth factor A. Nat Rev Mol Cell Biol.

[15] Varricchi G (2015). Angiogenesis and lymphangiogenesis in inflammatory skin disorders. J Am Acad Dermatol.

[16] Zhang ZG (2000). VEGF enhances angiogenesis and promotes blood-brain barrier leakage in the ischemic brain. J Clin Invest.

[17] Ferrara N (2004). Vascular endothelial growth factor: basic science and clinical progress. Endocr Rev.

[18] Hurwitz H (2004). Bevacizumab plus irinotecan, fluorouracil, and leucovorin for metastatic colorectal cancer. N Engl J Med.

[19] Klement G (2000). Continuous low-dose therapy with vinblastine and VEGF receptor-2 antibody induces sustained tumor regression without overt toxicity. J Clin Invest.

[20] Brown LF (1992). Vascular permeability factor mRNA and protein expression in human kidney. Kidney Int.

[21] Berse B (1992). Vascular permeability factor (vascular endothelial growth factor) gene is expressed differentially in normal tissues, macrophages, and tumors. Mol Biol Cell.

[22] Eremina V (2003). Glomerular-specific alterations of VEGF-A expression lead to distinct congenital and acquired renal diseases. J Clin Invest.

[23] Eremina V (2008). VEGF inhibition and renal thrombotic microangiopathy. N Engl J Med.

[24] Maynard SE (2003). Excess placental soluble fms-like tyrosine kinase 1 (sFlt1) may contribute to endothelial dysfunction, hypertension, and proteinuria in preeclampsia. J Clin Invest.

[25] Erpenbeck L (2016). ADAMTS13 endopeptidase protects against vascular endothelial growth factor inhibitor-induced thrombotic microangiopathy. J Am Soc Nephrol.

[26] Noris M, Remuzzi G (2009). Atypical hemolytic-uremic syndrome. N Engl J Med.

